# JNK-signaling: A multiplexing hub in programmed cell death

**DOI:** 10.18632/genesandcancer.155

**Published:** 2017-09

**Authors:** Danny N. Dhanasekaran, E. Premkumar Reddy

**Affiliations:** ^1^ Department of Cell Biology, Stephenson Cancer Center, University of Oklahoma Health Sciences Center, Oklahoma, OK, USA; ^2^ Department of Oncological Sciences, and Department of Structural and Chemical Biology, The Tisch Cancer Institute, Icahn School of Medicine at Mount Sinai, New York, NY, USA

**Keywords:** JNK, apoptosis, cell death, necroptosis, ferroptosis

## Abstract

Jun N-terminal kinases or JNKs have been shown to be involved in a wide array of signaling events underlying tumorigenesis and tumor progression. Through its interaction with a diverse set of signaling proteins and adaptors, JNKs regulate cell proliferation, invasive migration, therapy resistance, and programmed cell death. JNKs have been shown to play a role in apoptotic as well as non-apoptotic programmed cell death mechanisms including those of necroptosis, ferroptosis, pyroptosis, and autophagy. Most of the tumorigenic regulatory functions of JNKs can be related to their ability to module cell death via these programmed cell death mechanisms. JNKs stimulate or inhibit cell death in a context-dependent manner by stimulating the expression of specific genes as well as by modulating the activities of pro- and anti-apoptotic proteins through distinct phosphorylation events. This review summarizes our current understanding of the role of JNK in programmed cell death and its impact on cancer growth, progression, and therapy.

## INTRODUCTION

Jun kinase was identified almost 25 years ago, initially as a member of the pp54 microtubule associated protein-2 kinases [1, 2] and subsequently characterized as genotoxic- or cytotoxic-stress activated kinase that preferentially phosphorylated and stimulated the activity of c-Jun [3-6]. Mammalian JNKs are encoded by three distinct, but closely related JNK genes, *JNK1*, *JNK2*, and *JNK3*. These JNKs along with their multiple splice variants form a family of proline-directed protein kinases, collectively belonging to the superfamily of mitogen activated protein kinases [7-10]. Initially, JNKs were identified as stress activated protein kinases that are involved in the transactivation of c-Jun by phosphorylating the N-terminal Ser-63 and Ser-73 residues factors [2-5, 11]. Subsequent studies have shown that JNKs can also be activated by growth factors as well as inflammatory cytokines [6, 12-15]. JNK-mediated signaling involves a three-tier kinase signaling module, typically consisting of an upstream MAP3K, a middle MAP2K, and a down-most MAPK in which JNK forms the last rung of the ladder [16]. The signaling relay mediated by this kinase module results in the activation of JNK through the phosphorylation of the Thr- and Tyr-residues of the TXY motif of JNKs by the upstream dual specificity MAP2K, MKK4 or MKK7. Anti-apoptotic as well as pro-apoptotic signals activate JNKs via these kinase modules and JNKs, in turn, activate apoptotic signaling either through stimulating the expression of apoptotic genes via the transactivation of specific transcription factors in the nucleus or by directly modulating the activities of mitochondrial pro- and anti-apoptotic proteins through phosphorylation events in the cytoplasm.

Analyses of JNK-regulated pathways have shown that they play a crucial role in both cell proliferation and cell death [17-20]. It appears that balance between these two signaling inputs, often dictated by the cellular contexts, finally determines whether the cells are committed to proliferation or programmed cell death [10, 15, 16, 18, 21, 22]. More and more studies are pointing to dysregulation of cell death as a major causative factor in growth and therapy-resistance in many cancers. Role of JNKs in the regulation of cell death through apoptotic signaling has been investigated to a great extent in the past [16]. However, recent studies have shown that non-apoptotic cell death mechanisms, such as necroptosis, pyroptosis, ferroptosis, and autophagy also play a significant role in health and disease [23-30]. Cell death regulated by apoptosis has been classified into an extrinsic pathway initiated by death receptors such as those of TNF-α, TRAIL, and FAS-L and an intrinsic pathway primarily initiated by mitochondrial events [31, 32]. It has been shown that JNK-mediated signaling is involved in the regulation of both of these pathways [10, 15, 16]. While a similar extrinsic- and intrinsic-pathway based classification remains to be established in necroptosis, pyroptosis, ferroptosis, and autophagy, analysis of the recent findings from several laboratories indicate a key role for JNKs in programmed cell death mediated by these non-apoptotic mechanisms. This review analyzes our current understanding of the mechanisms through which JNK-signaling module regulates multiple modalities of programmed cell death which in turn impact cancer growth, chemotherapy, and therapy resistance.

### Programmed Cell Death: Apoptosis, Necroptosis, Ferroptosis, Pyroptosis, and Autophagy

Of the different modalities of programmed cell death, apoptosis has been well characterized whereas the presence and the significance of other modalities of cell death are being realized only now. Apoptosis has been shown to play a major role in physiological cell death, with specific biochemical and morphological characteristics. The salient feature of apoptosis is the activation of caspase family of proteases that act as the executioners of apoptosis [27, 31]. Morphologically, apoptosis is characterized by membrane blebbing and rounding up of the cells accompanied by cytoplasmic and nuclear volume decrease along with chromatin condensation and nuclear fragmentation. In contrast, necroptosis differs from apoptosis in which the necroptotic cells show ruptured cell membrane, cytoplasmic and cytoplasmic organelle swelling, and a moderate chromatin condensation [27]. It has been shown that apoptotic cytokines such as TNF triggers necroptosis when the apoptotic pathway is inhibited [27, 30]. In necroptosis, receptor-interacting Ser/Thr protein kinase 3 (RIPK3) acts as the executioner in this caspase-independent cell death pathway. In contrast to necroptosis, pyroptosis is a caspase-dependent programmed cell death pathway involving caspase 1 and/or caspase 5 [25, 30]. However, pyroptosis differs from apoptosis in that it induces plasma membrane pores, cell swelling, and osmolyis in a caspase-1-dependent manner. Ferroptosis differs from other modalities by the presence of condensed mitochondria, reduced mitochondrial cristae, and outer mitochondrial membrane rupture [27, 28]. Biochemically, it is characterized by the accumulation of lipid-based reactive oxygen species and iron. It appears that the subsequent membrane lipid damage and/or lipid peroxide inactivation of intracellular proteins induce cell death [33]. However, there is an alternative view that envisages ferroptosis as an inducer of cell death via autophagy [34]. In the case of autophagy mediated cell death, it is induced by the dedicated autophagy proteins such as Beclin-1 and ATG-family of proteins [30, 35, 36]. Either directly or indirectly JNKs have been shown to modulate all of the cell death modalities as discussed below.

### JNKs and Apoptosis

All the three JNKs have been shown to be involved in stimulating apoptotic signaling. The primary evidence that JNKs are involved in apoptotic signaling came from the observation that γ-ray stimulated the activation of JNK-1 along with an increase in DNA fragmentation in Jurkat cells [37]. These studies demonstrated that the γ-rays stimulate a delayed but persistent activation of JNK-1 in Jurkat cells with a concomitant increase in DNA fragmentation. Although these initial studies failed to establish a cause and effect relationship between JNK and DNA-fragmentation, the results indicated an underlying the differences between the growth factor-induced, anti-apoptotic JNK activation *versus* the γ-ray induced pro-apoptotic activation. While γ-ray induced JNK-activation was delayed, the growth factor induced JNK-activation was rapid and transient. Subsequent studies expanded the role of JNK in TNFα-, FasL -, X-ray-, and UV-ray-induced apoptosis in different cellular and physiological contexts. Unequivocal evidence that JNK1 and JNK2 are involved in apoptotic signaling came from studies using embryonic fibroblasts derived from JNK1−/−JNK2−/− mice that showed resistance to apoptosis in response to diverse genotoxic and cytotoxic stress [38, 39]. The defect in apoptosis can be correlated with the lack of mitochondrial depolarization, cytochrome C release, and the activation of caspases. Interestingly, these cells were responsive to Fas-mediated apoptosis suggesting that JNK1 and JNK2 are not required for the Fas-induced apoptosis of these fibroblasts.

**Figure 1:**
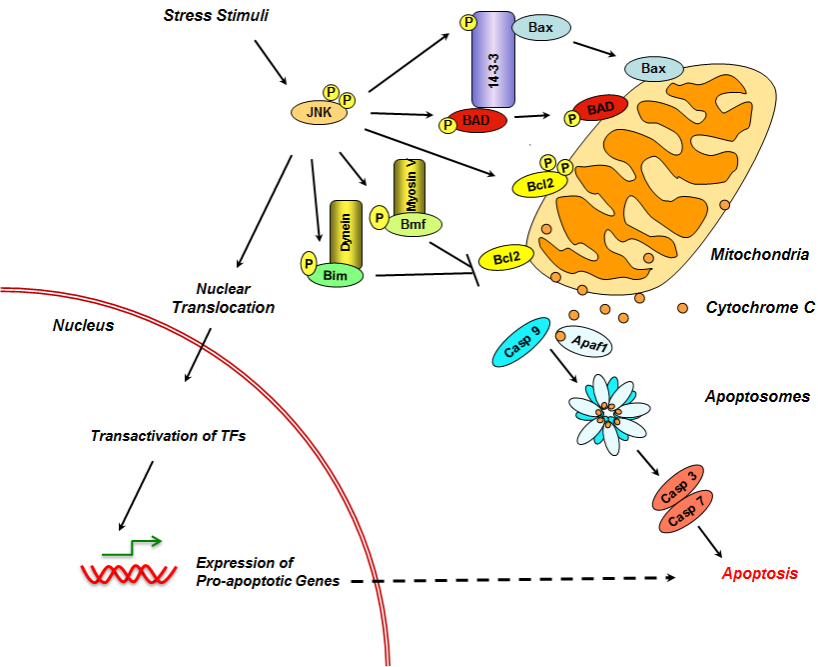
Role of JNK in Intrinsic Apoptosis JNK promotes intrinsic apoptosis by multiple mechanisms. In one mechanism, JNK stimulates the expression of apoptosis-specific genes through the transactivation of c-Jun and other target transcription factors (TF). In addition, JNK phosphorylates Bim and Bmf from their scaffold proteins to inhibit the anti-apoptotic activity of Bcl2. JNK also inhibit the activity of BCL2 through direct phosphorylation. JNK also promotes the translocation of Bad and Bax from their 14-3-3 mediated sequestering complex through direct phosphorylation. Translocated Bax and Bad stimulate the release cytochrome C (Cyt C) from the mitochondrial inner membrane. Released cytochrome C, in combination with Apaf-1 and caspase-9 form the apoptosomes, which triggers capase-9 cascade leading to the activation of the executor caspases, caspase-3 and -7 and apoptosis.

**Figure 2:**
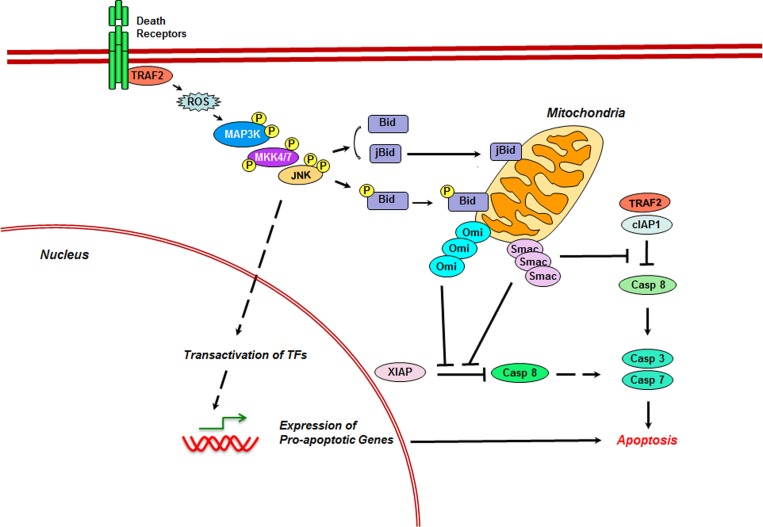
Role of JNK in Extrinsic Apoptosis In addition to stimulating the expression of death receptors, ligands, and pro-apoptotic proteins, death receptor-activated JNK promotes extrinsic pathway through the direct phosphorylation of Bid. Phosphorylation-mediated cleavage of Bid leads to the generation of jBid and its translocation to mitochondria. In mitochondria, both JNK-phosphorylated full-length Bid as well as jBid promotes the release of Smac and Omi. Inhibition of XIAP by Smac and Omi in addition to the inhibition of cIAP1 leads to the activation of the executioner caspases 3 and 7, leading to apoptosis.

Likewise, the apoptotic role for JNK3 in apoptosis was validated using JNK3−/− mice that showed resistance to glutamate-induced apoptosis of their hippocampal neurons [40]. A general mechanism through which JNKs modulate apoptotic pathway involves their ability to stimulate the expression of pro-apoptotic genes through the activation of specific transcription factors such as c-Jun, p53, and p73 [41-44]. In addition to their effect on gene expression, JNKs play an active role in the regulation of both the intrinsic (Figure 1) and extrinsic apoptotic pathways (Figure 2).

### Nuclear signaling of JNK in the regulation of apoptosis

Upon activation by the upstream MAP2Ks, the phosphorylated JNK translocates to the nucleus where it phosphorylates and transactivates c-Jun (8, 41) resulting in the formation of AP-1. AP-1 is involved in the transcription of a wide variety of proteins, including several pro-apoptotic proteins [16, 45] such as TNF-α, Fas-L, and Bak [46]. JNK can also phosphorylate several other transcription factors including JunD, ATF2, ATF3, Elk 1, Elk-3, p53, RXRα, RARα, AR, NFAT4, HSF-1, and c-Myc [47]. Thus, in the context of apoptosis, the nuclear activity of JNK can potentially lead to an increase in the expression of pro-apoptotic genes and/or a decrease in the expression of pro-survival genes. There is sufficient evidence that the nuclear activity of JNK and the transactivation of c-Jun are required for its apoptotic activity. It has been observed that JNK is required for the apoptosis of central nervous system neurons and the expression of dominant negative inhibitors of nuclear JNK confer resistance to their apoptosis following trophic support [48]. These findings suggest an important role of nuclear JNK in promoting apoptotic signaling. It is significant to note here that the non-phosphorylatable mutants of c-Jun confer resistance to apoptosis of MEFs in response to UV-irradiation. Together, these results may be indicative of a role for JNK-c-Jun/Ap1 mediated expression of pro-apoptotic genes in JNK-mediated apoptosis.

An alternate pathway contributing to JNK-mediated apoptosis involves the phosphorylation of the p53 family of proteins by JNK [49]. It has been suggested that the phosphorylation of p53 at Ser6 by JNK inhibits ubiquitin-mediated degradation of p53 and thereby stabilizing the levels of p53. Recent studies have shown that the phosphorylation of p53 at Ser6 by JNK2 is critically required for the apoptotic pathway [43]. It has been observed that the expression of FDH (10-formyltetrahydrofolate dehydrogenase) is drastically reduced in tumors and its elevation induces p53-dependent apoptosis. Further analyses of this apoptotic event indicated that FDH induces direct phosphorylation of p53 at Ser6 involving both JNK1 and JNK2. Treatment of FDH-expressing cells with JNK inhibitor SP600125 or silencing of JNK1/2 or inhibited the phosphorylation of p53 at Ser6 as well as p53-dependent apoptosis in response to DNA-damage. Since p53 is involved in the expression of several pro-apoptotic genes such as Bax (Bcl2-associated X protein) and PUMA (p53 up-regulated modulator of apoptosis), it is quite possible that apoptotic pathway activated by JNK involves p53-mediated upregulation of pro-apoptotic genes.

DNA damage that activates JNK also causes the stabilization and activation of p73, a member of the p53 family of transcription factors [44]. Similar to p53, p73 induces apoptosis by increasing the expression of pro-apoptotic genes including Bax and PUMA. Analysis of this pathway has shown that JNK is required for p73-mediated apoptosis induced by the DNA damaging agent cisplatin and it involves the phosphorylation of p73 at several serine and threonine residues. Consistent with the notion that JNK and its activity are required for p73-mediated apoptosis, mutation of JNK-phosphorylation site in p53 abrogates cisplatin-induced stabilization of p73 along with a marked reduction in p73- transcriptional activity, and cisplatin-induced apoptosis. Thus, JNK can stimulate the expression of pro-apoptotic genes and decrease the expression pro-survival genes via multiple transcription factors in cell-type and stimuli specific manner.

### JNK in Intrinsic Apoptotic Pathway

In the intrinsic pathway, DNA damage or cytoplasmic stress activates the pro-apoptotic proteins BAX or BAK via BH3-only protein. Activated BAX or BAK induces mitochondrial outer membrane permeabilization (MOMB), resulting in the release of cytochrome C along with other pro-apoptotic proteins such as SMAC/DIABLO and OMI/HTRA2 from the mitochondrial intermembrane space. Released cytochrome C, in combination with Apaf-1 and caspase-9, form the apoptosomes, which triggers the caspase-9 cascade leading to the activation of the executor caspases, caspase-3 and -7 and consequent cellular apoptosis. This process is further potentiated by SMAC and OMI that inhibits the anti-apoptotic protein XIAP. The observation that MOMB and the consequent release of cytochrome C in response to UV-irradiation were inhibited in JNK1−/− and JNK2−/− MEFs indicated a role for the kinases in the intrinsic pathway [38, 50]. One mechanism through which JNK mediates the release of mitochondrial cytochrome C is through its ability to increase the expression of BAX via transcriptional activation of c-Jun leading to MOMB [51, 52]. More importantly, JNK triggers the release of cytochrome C by stimulating the translocation of BAX to mitochondria [53]. JNK mediates the translocation of Bax through the phosphorylation of 14-3-3α and/or 14-3-3ζ protein, a cytoplasmic anchor protein for Bax. Phosphorylation of 14-3-3 proteins leads to the dissociation of BAX, following which it translocates to mitochondria to induce MOMB and cytochrome C release. In addition, JNK directly phosphorylates 14-3-3-associated Bad to promote its translocation to mitochondria and subsequent release of cytochrome C.

JNK promotes intrinsic apoptosis further by targeting BH3-only family of pro-apoptotic proteins [54]. During UV-induced apoptosis, JNK phosphorylates the pro-apoptotic BH3-only proteins Bim and Bmf to release them from sequestering dynein and myosin V motor complexes respectively [55, 56]. Bim and Bmf, thus released, can activate Bax and/or Bak to initiate apoptosis [54, 57, 58]. Furthermore, JNK-phosphorylated Bim can also bind and neutralize the anti-apoptotic activities of Bcl2 and Bcl2 homologs [59, 60]. JNK has also been shown to promote apoptosis through the phosphorylation of the pro-apoptotic protein Bad [61, 62]. JNK specifically phosphorylates the Ser128 of BAD and promotes its apoptotic effect of BAD in the primary granule neurons of the rat cerebellum [61]. In the absence of apoptotic stimuli, the pro-survival kinases Akt-1, PAK-1, and PKA inhibit the apoptotic activity of BAD by phosphorylating it at Ser112/136/155, following which the phosphorylated BAD is sequestered by 14-3-3 family of proteins. JNK promotes apoptosis in this context by phosphorylating Bad at Ser128, an event that inhibits Bad-14-3-3 interaction. JNK potentiates this mechanism further by phosphorylating 14-3-3 protein at Ser184 following which 14-3-3 releases the sequestered BAD [53, 63]. There is also evidence that JNK promotes apoptosis by directly phosphorylating Bcl-2 to inhibit its anti-apoptotic activity. During paclitaxel-induced apoptosis of breast cancer cells, it has been shown that JNK is activated and the activated JNK phosphorylates Bcl2 at Ser70 to induce apoptosis [64, 65].

Although it is not likely that all of these JNK-mediated events occur in a single cell type or physiological context, the findings discussed here highlight the multiple mechanisms through which JNK can trigger and/or potentiate extrinsic apoptotic pathway (Figure 1).

### JNK in Extrinsic Apoptotic Pathway

The extrinsic pathway is characterized by the apoptotic signaling initiated by the activation of the death receptors, namely TNFR1, DR3 (TRAMP), CD95 (Fas, APO-1), Trail-R1 (DR4), TRAIL-R2 (DR5), and DR6, by their respective ligands [65, 66]. Activated death receptors recruit the adaptor molecules such as FAS-associated death domain protein, which activates caspase 8. Activated caspase 8 directly activates caspase 3 and caspase 7 that initiates apoptosis. However, many of the death receptors also stimulate the activation JNKs. JNKs primarily contribute to the extrinsic pathway through AP1-mediated increased expression death receptors, ligands, and pro-apoptotic proteins such as Bak, Bim [67], Bax [68], Trail-R2 [69, 70]. Activated JNKs in these pathways are critically involved in the potentiation of the extrinsic pathway. In TNF-α induced apoptosis of HeLa cells, JNK-mediated phosphorylation leads to the cleavage of Bid. The 21 kDa cleaved fragment of Bid translocates to mitochondria and selectively promotes the release of SMAC/DIABLO and OMI, two of the mitochondrial intermembrane proteins, that can inhibit the anti-apoptotic protein, XIAP, an inhibitor of caspase 8. In addition, OMI inhibits cellular-inhibitor of apoptosis protein 1 or cIAP1, another inhibitor of caspase 8 [71]. Inhibition of XIAP and cIAP1 leads to the activation of the executioner caspases 3 and 7, leading to apoptosis [72]. Even in the absence of such a cleavage, JNK-phosphorylated full length Bid translocates to mitochondria and potentiates extrinsic signaling during TNF-induced apoptosis in PC12 cells [73]. Cumulatively, the multiple signaling inputs generated by JNK promotes extrinsic apoptotic pathway (Figure 2)

### JNK in Necroptosis

While different stimuli can induce necroptosis including DNA-damage and activation of death receptors such as TCR, TLR or TNFR [74]. Necroptotic stimuli, through cell type and stimulus dependent specific protein-protein interactions, activate the Receptor-Interacting serine/threonine Protein Kinase or RIP3. Activated RIP3 and the downstream pseudokinase Mixed-Lineage kinase domain Like (MLKL) oligomers form a necrosome complex and translocate to mitochondria-associated endoplasmic reticulum. RIP3-dependent phosphorylation of the mitochondrial proteins PGAM5 and Drp-1 leads to mitochondrial fission and ROS production [75]. ROS, thus generated, has been proposed as an executioner of necroptosis. In murine fibroblasts, it has been shown that the ROS, thus generated, induces necroptosis through the oxidative inactivation of MAP kinase phosphatase 1, which downregulates the activity of JNKs. Sustained activation of JNK, resulting from the inactivation of MKP1, is proposed to induce cell death via cytochrome C release or phosphorylation of BID [76]. Recent studies using 2-methoxy-6-acetyl-7-methyljuglone (MAM)-treated colon cancer cells further validated a role for JNK in MAM-induced necroptosis [77]. Treatment of HCT116 and HT29 colon cancer cells with MAM induces necroptosis marked by mitochondrial depolarization, ATP depletion, and an increased mitochondrial reactive oxygen species (ROS). It was observed that MAM-induced RIP1/RIP3 complex triggered necroptosis in these cells through cytosolic calcium (Ca^2+^) accumulation, sustained c-Jun N-terminal kinase (JNK) activation, and increased mitochondrial ROS levels. Both calcium chelator and JNK inhibitor could attenuate necroptotic features, including mitochondrial ROS elevation, mitochondrial depolarization, and ATP depletion. These results point out Ca2+ accumulation, and JNK-activation are upstream of mitochondrial depolarization and increased ROS levels. It was also observed that the quenching of mitochondrial ROS with the overexpression of manganese superoxide dismutase (MnSOD) protected the cells from necroptotic cell death, indicating the crucial role of mitochondrial ROS in MAM-induced necroptosis. Treatment of cells with the mitochondrial complex II inhibitor, 2-thenoyltrifluoroacetons, reversed MAM induced mitochondrial ROS generation as well as cell death, indicating the complex II was the ROS-producing site.

**Figure 3:**
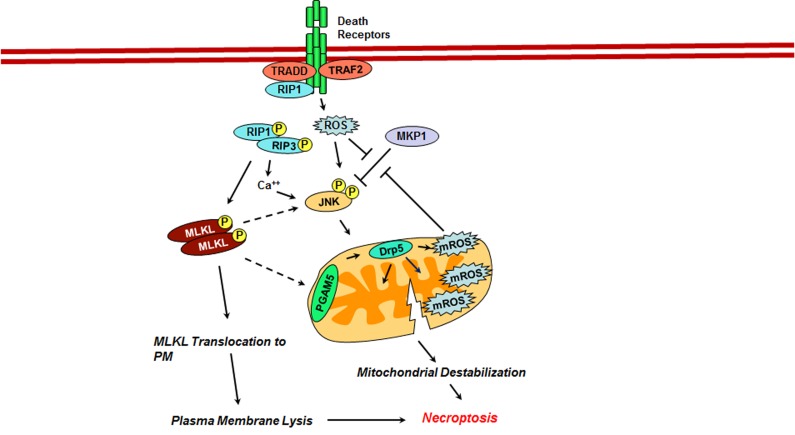
Role of JNK in Necroptosis JNK promotes necroptosis through a mitochondria-dependent mechanism. Diverse cytotoxic and genotoxic stress stimuli and the activation of death receptors lead to the activation of RIP3K, which in turn potentiates and sustains the activation of JNK through MLKL as well as Ca^++^-dependent mechanisms. An alternate mechanism for the sustained activation of JNK involves the inhibition of MKP1 by ROS generated by the death receptor signaling. Activated JNK promotes mitochondrial dysfunction including mitochondrial ROS generation and mitochondrial destabilization through a mechanism that remains to be clarified.

Although these studies have validated the notion that mitochondrial ROS acts as an executioner in necroptosis, it has also been observed that necroptotic death can be executed by a mitochondria-independent mechanism. In HEK293 cells, it was observed that TNF-induced necroptosis induced RIP3-mediated phosphorylation of MLKL and subsequent homotrimerization of MLKL. MLKL-homotrimer translocates to the plasma membrane and promotes the breakdown of plasma membrane presumably through its interactions with cationic channels that facilitates Ca^2+^ influx [78]. However, it is not clear whether JNK activated by TNF- plays a role in this pathway. Thus, it appears that the sustained activation of JNK promotes necroptotic cell death via mitochondrial ROS or through interfacing with the components of the intrinsic apoptotic pathway (Figure 3).

### JNK in Pyroptosis

**Figure 4:**
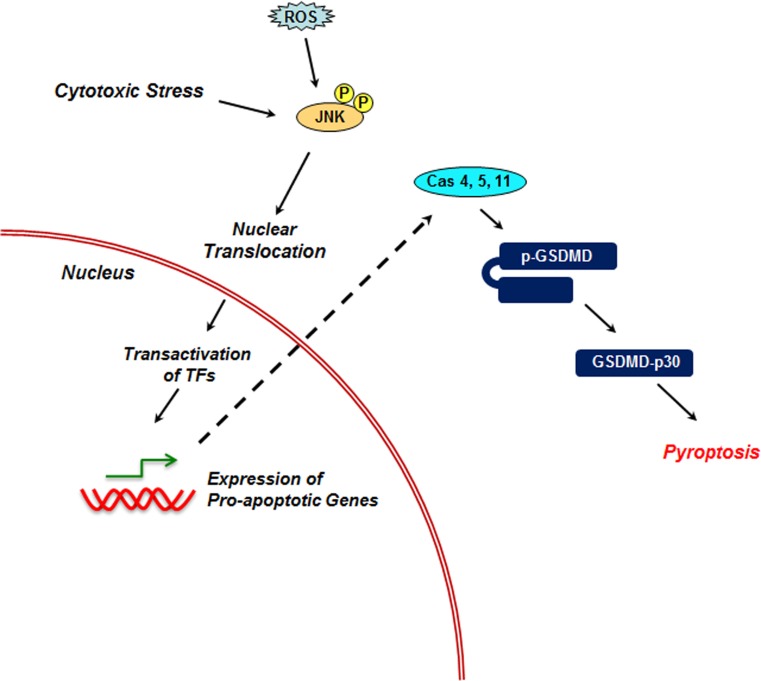
Role of JNK in Pyroptosis In non-canonical pyroptotic pathway, cytotoxic stress activated JNK stimulates the expression of Caspase 11 that cleaves the pro-gastermin-D p-GSDMD) into an active N-terminal (GSDMD-p30) and a C-terminal fragment. GSDMD-p30 induces pyroptosis through the formation of pyroptotic pores.

Pyroptosis is an inflammatory caspase-dependent cell death mechanism characterized by pore formation in the plasma membrane accompanied by relatively low intensity DNA-damage, and ADP-ribose polymerase activation. Caspase 1, 4, 5, and 11 constitute the family of inflammatory caspases that can be activated by inflammatory agents such as double-stranded DNA and bacterial toxins [25, 30]. In a canonical pyroptotic signaling pathway, the inflammatory stimuli lead to the direct activation of caspase. Caspase 1, in turn, cleaves the 54kDa protein known as gastermin D (GSDMD), following which the N-terminal fragment of gastermin-D forms oligomers that form pore in the plasma membrane and other pyroptotic events leading to cell death [79]. Caspase 1 has also been shown to cleave Caspase 3 and 7 independent of gastermin 2 cleavage [80]. In the non-canonical pyroptotic signaling pathway, the inflammatory agents such as bacterial lipopolysaccharides activate caspase 4/5/11, which in turn cleaves GSDMD, releasing the pyroptotic N-terminal fragment of GSDMD, known as GSDMD-p30 [81, 82]. Although the precise mechanism remains to be elucidated, it has been shown that the GSDMD-p30, thus formed, associates with plasma membrane as well as mitochondrial membrane and induces the formation of pyroptotic pores [83]. JNK-signaling appears to play a role in the non-canonical pyroptotic signaling pathway [25]. It has been shown that the infection of bacterial pathogen in bone marrow derived macrophages leads to the increased production of ROS, ROS-induced activation of JNK1/2, and Subsequently, JNK-mediated increased expression of caspase 11 [84]. As discussed above, increased in the levels of caspase 11 could trigger pyroptotic cell death via GSDMD cleavage and the generation of GSDMD-p30. Such an increase in ROS following inflammatory stimuli has also been observed in lipopolysaccharide-induced caspase 1-dependent pyroptotic cell death in human umbilical vein endothelial cells [80]. Although the role of JNK in this cellular context was not assessed, it is possible that the increased ROS leads to the activation of JNK and JNK-mediated potentiation of non-canonical pyroptotic signaling via caspase 11 (Figure 4). Thus, JNKs appears to play a potentiating role in the regulation of pyroptosis.

### JNK in Ferroptosis

Ferroptosis as a mechanism of programmed cell death was identified with the use of erastin or RSL, a specific Ras inhibitors that induced cell death to HRasV12 expressing cells compared Ras-WT expressing cells [33, 85, 86]. Analysis of mechanism underlying the cell death triggered by these molecules indicated that it involves the activation of a non-apoptotic signaling pathway dependent on cellular iron and lipid-based reactive oxygen species [27, 28, 33]. Most of our understanding of ferroptosis comes from the analysis of cell death pathway induced by erastin and RSL. Using cells in which specific apoptotic or necroptotic signaling components are suppressed, the distinct mechanism underlying ferroptosis has been identified. A variety of intrinsic and extrinsic factors induce ferroptosis in diverse cellular and physiological contexts. Essentially, ferroptotic cell death is induced by the accumulation of lethal lipid-based reactive oxygen species (L-ROS) generated by iron-driving lipid peroxidation by iron-containing enzymes such as heme oxygenase-1[28] or arachidonate 15-lipoxygenase-1 [87]. Although relatively little is known about the physiological role of this pathway, recent studies have shown that the lipid repair enzyme glutathione peroxidase 4 acts as a gatekeeper to inhibit this process whereas many of the ferroptotic stimuli converge on inhibiting this enzyme to promote the accumulation of L-ROS and resultant ferroptosis. A physiological role for ferroptosis has been established by the observation that p53-mediated tumor suppression may involve ferroptosis [88]. Considering the multiple mechanisms through which JNKs regulate the levels and activity of p53, it is possible that JNK, albeit indirectly, is involved in the regulation of ferroptosis. More direct involvement of JNK in ferroptosis was observed in studies using the JNK-inhibitor, SB600125, in HL60 cells [89]. In these cells, erastin induces the activation of JNK and p38MAPK and the inhibition of these kinases using the respective inhibitors protected the cells from ferroptotic cell death. Thus, it appears that kinase activity of JNK and p38MAPK is required for erastin-induced ferroptosis. However, the mechanism by which these kinases, especially, JNK module ferroptotic cell death remains to be identified.

### JNK in autophagic cell death

**Figure 5:**
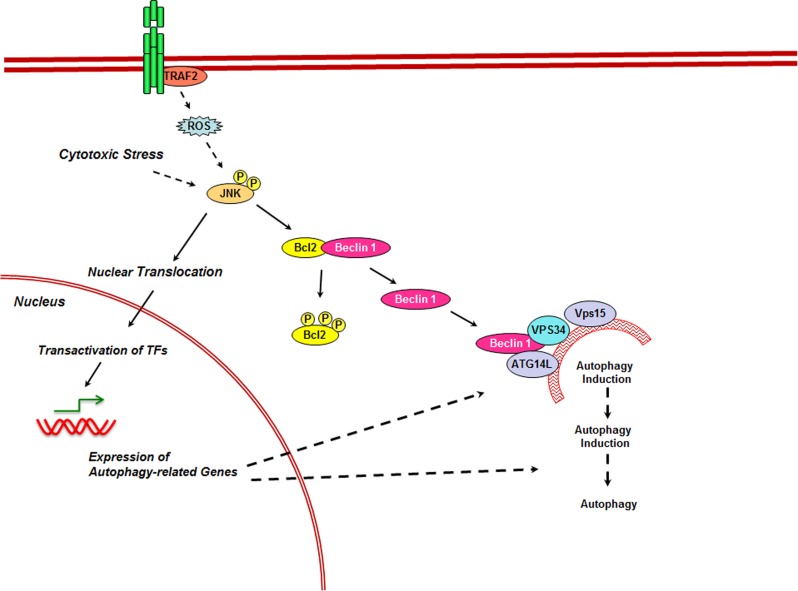
Role of JNK in Autophagy Intrinsic cytotoxic stress stimuli as well as the stimuli from the death receptors such as TNFR stimulates the activation of JNKs either directly or through the generation of ROS. Activated JNK promotes autophagy at two cellular locales. In the nucleus, through the transactivation of FoxO an AP1 family of transcription factors, JNK stimulates the expression of several autophagy related genes including ATG5, ATG7, LC3, and Beclin 1. In the cytoplasm, activate JNK directly phosphorylates BCL2 to release the sequestered Beclin 1. Released Beclin 1 associates with other autophagy proteins Vps15, Vps34, and ATG14L to induce autophagy.

Autophagy is a lysosome-dependent process in which the cytoplasmic components of the cells are sequestered in the double membrane autophagic vesicles or autophagosomes and fuse with lysosomes forming autolysosomes in which the cargo of the autophagosome is degraded [90-92]. Although autophagy was initially identified as a pro-survival mechanism during nutrient-starvation, it is being increasingly realized that autophagic cell death (ACD) forms a major non-apoptotic cell death mechanism regulated by JNK. JNK regulates ACD at multiple levels involving both nuclear and cytoplasmic events (Figure 5). In the nucleus, JNK has been shown to upregulate the expression several autophagy related genes in response to specific death promoting stimuli. JNK-activation and JNK-mediated expression of autophagy related genes (ATG) have been shown to be required for the activation of ACD by caspase 8 inhibitor [93], TNFα [94], ceramide [95, 96], bufalin [97], and H-Ras [98] in different cell types. The genes upregulated by JNK includes the ATG genes, ATG5, ATG7, LC3, and Beclin1 [35, 36]. In addition to these ATG genes, JNK is also involved in the increased expression of *DRAM* or Damage Regulated Autophagy Regulator, a p53-target gene [99]. In Ewings Sarcoma cell lines, 2-methoxyestradiol induces ACD. It has been shown that 2-ME induced autophagy requires the activation of JNK and JNK-mediated upregulation of DRAM expression [99, 100]. By virtue of its ability to transactivate c-Jun, JNK-mediated upregulation of autophagy related genes involves AP1 transcription factor complex. In addition, JNK promotes the increased expression of autophagy related genes through O-subclass of the forkhead box family of transcription factors (FoxO), a major regulator of ATG-gene expression. JNK stimulates the activity of FoxO transcription factors through direct phosphorylation [101, 102]. FoxOs, thus stimulated, upregulate the expression of multiple ATG genes [103].

JNK also induces autophagic cell death independent of its role in enhancing the expression of autophagy related genes. It has been well-documented that the anti-apoptotic protein BCL2/BCL-xL complexes and sequesters Beclin1 and inhibit cellular autophagy [104, 105]. During ACD response, activated JNK phosphorylates BCL2/BCl-xL so as to release Beclin 1 from the BCL2-Beclin 1 complex and promote autophagy [106, 107]. It has been proposed that the sustained phosphorylation of BCL led to Beclin 1-mediated autophagic cell death [108]. Similarly, JNK reverses the BIM mediated inhibition of autophagy involving the sequestration of beclin 1 to microtubules [109]. It has been observed that dynein light chain mediates BIM and Beclin 1 interaction that inhibits Beclin 1 regulated autophagosome formation. Activated JNK phosphorylates BIM, releasing Beclin 1 to induce autophagy [109].

## CONCLUSIONS

The role of JNKs in the regulation of different modalities of cell death summarized here indicates the pervasive role of JNKs in regulating pathways related to cell death. Of the different modalities of cell death, the role of JNKs is well characterized in apoptosis whereas, the role of JNK in other modalities of cell death is beginning to be realized only now. Although the physiological significance of necroptosis, pyroptosis, ferroptosis, and autophagic cell death in normal cellular homeostasis is not fully understood, the observation that all of these pathways can be directly or indirectly activated by JNK points to the possible cross-talk within the signaling nodes regulated by JNK. Taking the analogy from our understanding of the role of JNK-mediated apoptosis in cisplatin resistance [110], it is possible that the interrogation of these signaling nodes could unravel novel therapeutic targets in cancer.
